# Emergence of Two Porcine Variants of Human Coxsackievirus B5 and B4 in the 20th Century That Caused Swine Vesicular Disease: A Retrospective Review

**DOI:** 10.3390/pathogens15060565

**Published:** 2026-05-23

**Authors:** Natalia F. Lomakina, Simone E. Adams

**Affiliations:** 1 The Gamaleya National Center of Epidemiology and Microbiology of the Russian Ministry of Health, Moscow 123098, Russia; 2 Department of Veterinary Medicine, University of Cambridge, Madingley Road, Cambridge CB3 0ES, UK; sea65@cam.ac.uk

**Keywords:** *Enterovirus betacoxsackie*, swine vesicular disease virus, RNA-virus recombination, emerging diseases

## Abstract

In this review, we examine the occurrence of two independent, single recombination events which occurred between human enteroviruses (*Picornaviridae*, *Enterovirus*, *Enterovirus betacoxsackie*). These recombination events contributed to the emergence of two viruses which adapted to pigs. These viruses have caused epizootics of swine vesicular disease (SVD) for many years. As was shown previously, the classical SVD virus (SVDV-1) originated from human coxsackievirus B5. The strain T75 (SVDV-2) emerged from human coxsackievirus B4 in the Tambov region of Russia, where it circulated from 1975 to 1977. A high percentage of similarity between both types of the SVD virus was found in the 3D protein coding region (88%). In our previous work, analysis of the VP1 gene dates the appearance of the SVDV-2 precursor to between 1954 and 1975. In this work, the origin of the genome region encoding non-structural proteins was analyzed and is believed to be a result of multiple recombination events between human enteroviruses (hypothetically, E1, E9, E11 and coxsackievirus A9). The recombination breakpoint between the region of structural CVB4 proteins and non-structural T75 proteins is located in region 2A. This mini-review also represents the historical research of SVDV-1 and SVDV-2 strains (O72(USS/6/72) and T75, respectively) isolated in the former Soviet Union.

## 1. Introduction

Swine vesicular disease virus (SVDV) (family: *Picornaviridae*; genus: *Enterovirus*; species: *Enterovirus betacoxsackie*) affects exclusively domestic pigs and wild boars, with both mild and severe forms of disease. It can also present sub-clinically with a prolonged carriage of the virus. Swine vesicular disease (SVD) is a highly contagious, but typically not fatal, infection [1], with clinical signs similar to more serious foot-and-mouth disease, vesicular exanthema, vesicular stomatitis, and senecavirus A disease.

Since January 2015, SVD has been removed from the World Organisation for Animal Health (WOAH) list of declared diseases [2]. However, in some SVD-free countries, the disease remains notifiable at the regional level. Current evidence indicates that Europe, Africa, the Americas and Oceania are free of SVD, but the disease is likely still present in various parts of eastern Asia.

Within the genus *Enterovirus*, the species *Enterovirus betacoxsackie* comprises a diverse group of viruses that infect humans and primates [3,4]. Among them are coxsackieviruses (CVs), known as CVB1-CVB6 and CVA9, echoviruses and many others. Frequent genome recombination events occur between these viruses, for which humans are the sole host [5,6,7,8,9]. Recombination is an important survival strategy and mechanism for the formation of new virus variants, contributing to the spread and expansion of the host range. SVDV is hypothesized to have a monophyletic origin from a common ancestor, arising via recombination between human CVB5 and other members of the Enterovirus B species, followed by host adaptation to swine [10,11,12,13]. Now, based on the antigenic cross-reactivity and the sequence homology between CVB5 and SVDV, SVDV is considered as a porcine variant of CVB5 [1,2,10,11,12,13,14].

Historically, SVDV was considered to have a single serotype [1]. Then, in 1975, Russian scientists discovered an unknown enterovirus which caused an epizootic that affected about 24,500 pigs with SVD-like clinical signs. Initially, the virus was identified as SVDV serotype 2 (SVDV-2), but much later, in the 21st century, it was determined that this virus was derived from coxsackievirus B4 (rather than CVB5) following multiple recombination events [14,15], making this the second case of interspecies transmission of a human betacoxsackievirus to pigs. Here, we refer to SVDV-1 and SVDV-2 as viruses derived from CVB5 and CVB4, respectively, while sometimes SVDV refers to SVDV-1 only.

This mini-review is devoted to the history and characterization of SVDV-2. It evaluates research which has been carried out among scientists and institutions around the world.

## 2. Methodology and Techniques in SVDV Research

SVDV is routinely isolated and propagated in IBRS-2 cells or another suitable porcine cell line. For laboratory detection and disease confirmation, immunological, serological, and RT-PCR tests are recommended and described in detail by WOAH [1].

Earlier studies from the 20th century utilized complement fixation and virus neutralization tests to detect viral antigens or antibodies against SVDV in samples, but these assays needed several days to yield results. Faster, enzyme-linked immunosorbent assays (ELISA) were developed in the 1990s, which significantly improved diagnostics [1]. This advance was achieved when monoclonal mouse antibodies (MAbs) were obtained against SVDV, which could then be used for diagnostics, as well as for research [1,16,17]. Notably, the 5B7 MAb has been widely used in both simple sandwich and in competitive serological ELISAs for the detection of viral antigens [1], while a panel of MAbs allowed for the differentiation of antigenic groups of SVD isolates [17].

The MAb-based ELISA, which was developed by Brocchi and colleagues [17], is briefly described here. Microplates are coated with a rabbit anti-SVDV immune serum to capture the virus particles from tested samples and the control reference strains. Then, MAbs specific to the reference strain of each antigenic group are added to the plates followed by anti-mouse secondary antibodies conjugated to horseradish peroxidase. The signal is then developed using a color enzyme reaction (for example orthophenylenediamine and substrate H_2_O_2_). Optical density is measured spectrophotometrically, and results are expressed as a percentage relative to the corresponding reference virus for each MAb within the panel.

There have also been advances in techniques for laboratory research. The first methods of complete genome sequencing were labor-intensive and time-consuming [14]. Therefore, for phylogenetic analysis, only short-length regions of important genes were sequenced by the Sanger method [11,17]. This was until next-generation sequencing, using the Illumina platform (Illumina, San Diego, CA, USA), was developed to quickly obtain complete genome sequences [12].

Nucleotide and amino acid sequence homology or diversity, as well as sequence alignment and building of phylogenetic trees for small datasets can be carried out by different programs such as MegAlign (Lasergene Software, v6, DNASTAR Inc., Madison, WI, USA) and MEGA 6. The latest versions of MEGA (https://www.megasoftware.net/) and BEAST 2 [18] are suitable for more precise phylogenetic analysis, evolutionary tree reconstruction, and molecular clock analysis.

According to the literature [5,19], recombination analysis for enteroviruses may be carried out by the methods detailed in the Recombination Detection Program [20], and the Genetic Algorithm for Recombination Detection [21] which are available on the Datamonkey website (https://www.datamonkey.org/gard, accessed on 15 April 2026; [22]), along with SimPlot software [23]. MEGA 6, BEAST 1.7.5 and SimPlot version 3.5.1 (S.C. Ray, 1997–2003) were used for phylogenetic analysis in the current work.

## 3. Genome Structure

Similar to other members within the enterovirus genus, SVDV has a single-stranded, polyadenylated, positive-sense RNA genome of 7400–7500 nucleotides (n.) with non-translated regions at the 5′ and 3′ ends. The 3′ region of the genome encodes structural proteins, while the 5′ region encodes non-structural proteins. Virion RNA is translated as a single polyprotein, which is first cleaved into three precursors (P1, P2, and P3), and subsequently into 11 mature proteins [24]. The structural proteins (VP1/1D, VP2/1B, VP3/1C, and VP4/1A (previous/current protein nomenclature)), which form the virion capsid, contain epitopes that induce antibody production and are recognized by neutralizing and monoclonal antibodies [10,17,25,26,27]. Non-structural proteins (2A, 2B, 2C, 3A, 3B, 3C, and 3D) include viral proteases (2A, 3C) as well as proteins involved in viral reproduction. 3D is an RNA-dependent RNA polymerase, which is responsible for RNA replication and transcription. Due to the lack of proofreading activity of this enzyme, mutations and genome recombination events are possible.

## 4. Outbreaks of Swine Vesicular Disease

SVD was first reported in Lombardy, northern Italy, in 1966 [28] where it remained endemic until 2015, when the last outbreak was recorded there [19,29]. Wild boars have been considered as carriers of the infection. Some researchers [11,17] propose that SVD has always been endemic to Southern China (including Hong Kong) and that the virus likely evolved in Southeast Asia [11]. From there, it is hypothesized that SVDV was repeatedly introduced into Europe, resulting in sporadic outbreaks.

From 1970 to 1983, SVD spread to many countries of the Eurasian continent (Hong Kong, Bulgaria, Austria, Great Britain, Italy, Poland, Japan, Romania, Germany, France, Malta, the Netherlands, Belgium). The disease then subsequently re-emerged in the Netherlands (1992), Spain (1993), Taiwan (1997, 1998, 2000), and Portugal (1995, 2003, 2004) [30].

For a long time, information about the presence of SVD in the former Union of Soviet Socialist Republics (USSR) was restricted and detailed only in documents for limited use. Most of the research conducted during this time was either unpublished or published only in internal proceedings of Soviet veterinary institutions [31,32,33].

Nevertheless, the first documented outbreaks of SVD occurred in February 1972 in the Odessa region (Tatarbunarsky district) of Ukraine and in Moldova. Then, from 1973 to 1976, outbreaks of SVD spread and were reported around the USSR in the Odessa, Kaliningrad, and Chelyabinsk regions, as well as the Khabarovsk and Krasnoyarsk Territories [32,33]. The antigenic properties of the pathogen were shown to be similar to European SVDV strains (Italy/66 and France/73).

In February 1975, a disease with vesicular syndrome affecting pigs in the Rasskazovo village of the Tambov region was identified. During 1975–1977, the epizootic spread to the Tambov, Voronezh, Saratov, and Moscow regions. Clinically, the disease had similar symptoms to SVD, but the pathogen was antigenically different from the SVD viruses discovered earlier [34].

From those epizootics, only three strains have been preserved in the laboratory and were designated O72 (Odessa region), T75 and T77 (Tambov region). The O72 strain was antigenically similar to SVDV strains collected from Italy/66 and France/73 [32], while the T75 and T77 strains had no commonality with SVDVs known at that time. Electron microscopy, together with analysis of physical and chemical properties of the virus, classified T75 as an enterovirus [35]. Cross-infection of pigs with the O72 and T75 strains showed that these viruses were distinct pathogens with similar clinical signs, as infection with each virus did not protect the animals after recovery and hyperimmunization with the other strain [36]. Similar results were obtained during experimental vaccination of pigs with vaccines prepared from strains O72 or T75. Each vaccine only protected against challenge with the homologous strain, no cross-protection was observed [37]. Although both viruses were assigned to the genus Enterovirus, they were classified as different SVDV serotypes due to the absence of antigenic cross-reactivity [36,37].

It is of note that both the O72 and T75 strains were able to replicate in pigs and in swine-origin IBRS-2 cells (a porcine kidney epithelial cell line), which have been recommended by WOAH [1]. It was later shown that both strains also grew well in human rhabdomyosarcoma (RD) cell culture [14].

## 5. Antigenic Analysis

**Figure 1:**
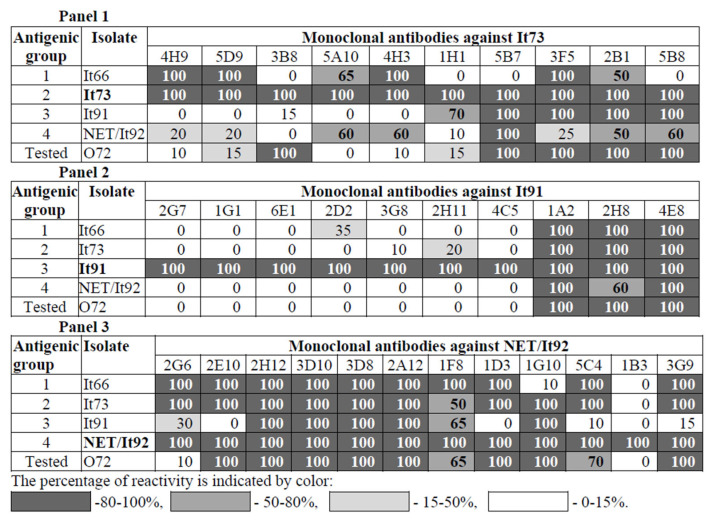
Reactivity patterns of swine vesicular disease viruses (SVDVs) with three panels of anti-SVDV monoclonal antibodies (MAbs). Reactivity of viruses with antibodies was measured by a capture ELISA as described in Section 2. The results are based on optical density values and are expressed as a percentage relative to the reference virus against which the panel was raised. The O72 strain was tested in comparison with the reference strains for each of the four antigenic groups: Italy/66 (It66), Italy/73 (It73), Italy/91 (It91), the Netherlands/Italy/92 (NET/It92). The SVDV reference strain for each Mab panel is given in bold. The reactivity percentage value is indicated in each cell of the panel and is highlighted according to value range. Analysis was carried out in 1998 in IZSLE [40].

In 1973, Graves [38] showed that SVDV was closely related to human CVB5 antigenically, but CVB5 and SVDV can be distinguished by various methods [10]. Progress in the study of SVDVs has mostly been achieved using monoclonal antibodies [17,25,26], and through sequencing and nucleotide analysis [11,39]. Italian scientists (IZSLE, Brescia, Italy) [16,17], in collaboration with colleagues from UK and Germany, characterized the 77 SVD isolates by antigenic and genetic methods including phylogenetic analysis for the 1D/VP1 nucleotide region. They identified four antigenic groups among the SVDV isolates observed in Europe from 1966 to 1994, as well as in early isolates from Japan and Great Britain (1972–1976). The first group was made up of the earliest and only examined strain, Italy/66 (It66), which was isolated in Italy in 1966; the second group consisted of viruses present in Europe and Japan between 1972 and 1981 with the reference strain Italy/73 (It73). The third group contained strains which were isolated during outbreaks of SVD in Italy between December 1988 and June 1992, with the reference strain Italy/91 (It91). A feature of this group was the absence of an epitope for the 5A10 MAb (Figure 1, panel 1). Finally, the fourth group included isolates from Romania, the Netherlands and Spain, which were recorded from 1987 to 1994 (the reference strain was Netherlands/Italy/92 (NET/It92)). The MAb marker for fourth group was 1B3 (Figure 1, panel 3). Three panels of MAbs were developed [17] and each panel included MAbs obtained against the reference strain of a known antigenic group (Figure 1).

All SVDV antigenic groups had antigenic cross-reactivity with human CVB5. Epitope mapping using SVDV-specific MAbs revealed that the epitopes were located primarily in VP1/1D, VP2/1B, and VP3/1C capsid proteins [25,26].

The O72 and T75 strains, which were isolated in the former USSR, were also tested by Brocchi and Borrego (performed in IZSLE, Italy, Brescia, 1998; published here for the first time). In preliminary experiments by double-sandwich ELISA with rabbit serum antibodies against CVB5, positive reactivity was found for the O72 strain, but results were negative against T75. These results were consistent with data obtained earlier, in Russia, which demonstrated an absence of a relationship between T75 and O72 by cell-culture-based neutralization assays [34,36,37].

Subsequent characterization of the O72 strain by the MAb-based ELISA with the panels of MAbs (Figure 1), as described in Section 2, assigned this virus to antigenic group II. The isolates within this group differ significantly in their antigenic properties from the later isolates within groups III and IV.

In Russia, the O72 (antigenic group II) and Italy/2008 (antigenic group IV) strains have been used in laboratory diagnostics for serological monitoring of SVDV in pigs imported from other countries [41,42]. Further, Russian researchers determined that the T75 strain belonged to the SVDV-2 serotype, due to the lack of antigenic relatedness to previously characterized SVDV strains [34,36,37].

## 6. Sequencing

At the end of the last century, the O72 strain was kindly transferred from the collection of FGBI “ARRIAH” (Russian Federation, Vladimir) to the Pirbright Institute (United Kingdom, England), where it was later sequenced completely and named USS/6/72 (GenBank: KT284982) in 2015 [12].

Before that, in 1998, the RNA-dependent RNA-polymerase genes (3D) of both the O72 and T75 strains (GenBank: AJ245863 and AJ245864) were sequenced in Russia. Comparative analysis using BLAST (https://blast.ncbi.nlm.nih.gov/Blast.cgi, accessed on 15 April 2026) showed a high percentage of similarity between the 3D genes from T75, O72, other SVDVs, and a partial fragment of human coxsackievirus A9 (CVA9). Unfortunately, the sequence of CVA9 (GenBank: DQ388926) was incomplete and limited to the 3CD region. Despite this, the observation of high similarity encouraged us to search for relatives or precursors of T75 among human enteroviruses.

**Figure 2:**
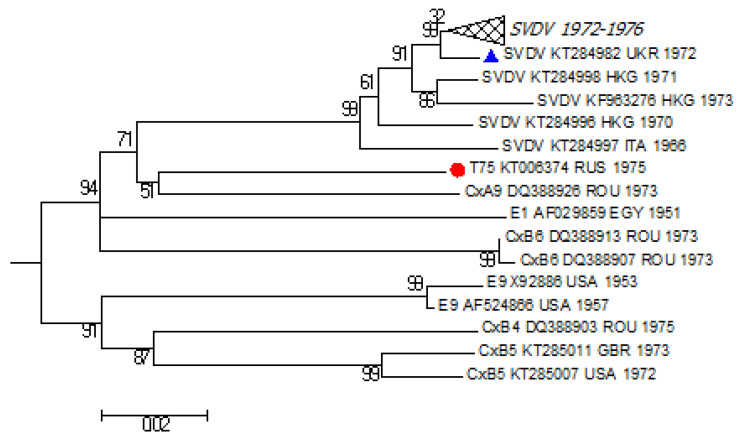
Phylogenetic tree showing the evolutionary relationships of enteroviruses using the 3CD region of the P3 precursor (fragment of 599 n.). The evolutionary history was inferred using the neighbor-joining method. The confidence probability (multiplied by 100), as estimated using the bootstrap test (1000 replicates), is shown next to the branches. The tree is drawn to scale (0.02), with branch lengths in the same units as the evolutionary distances used to create the phylogenetic tree. The evolutionary distances were computed using the Tamura 3-parameter method and are in the units of the number of nucleotide substitutions per site. The analysis involved 45 nucleotide sequences, but only the subtree is shown. Evolutionary analyses were conducted in MEGA 6 [43]. Triangle indicates O72 strain of swine vesicular disease virus (SVDV-1) isolated in the Ukraine in 1972. Circle indicates T75 strain of SVDV-2.

The complete genome sequence of T75 (GenBank: KT006374) was determined much later, in 2015 [14]. Surprisingly, the T75 strain had similarity with human CVB4 in the structural region, which is responsible for receptor binding and immunogenicity. As CVA9 had a high degree of similarity with T75 in the 3CD region, we aimed to understand more about the homology and phylogeny of these viruses in comparison with other similar viruses. The 3CD region (KT006374, fragment 5844-6459) showed a 90% similarity to CVA9 (GenBank: DQ388926), which was first discovered in Romania in 1973 (isolate 113/73/2 [6]). On the phylogenetic tree, T75 3CD occupies an intermediary position between SVD viruses and human enteroviruses (Figure 2). Unfortunately, as the complete genome sequence for the CVA9 strain is not available, further analysis of other genes could not be performed.

Phylogenetic analysis of the VP1 region was done both for O72 and T75 strains using a Bayesian likelihood-based algorithm implemented in Beast version 1.7.5. Strain O72 shared 87.3–97% identity with other SVDVs derived from CVB5, while the strain T75 shared 80.0–90.4% identity with CVB4 sequences available in GenBank (Table 1) [14]. Therefore, it was determined that T75 belongs to the CVB4 type, as viruses sharing >75% nucleotide sequence identity in the VP1 genome region belong to the same enterovirus type [30,44].

## 7. Recombination Analysis

Although T75 was clearly identified as a recombinant virus, exactly which viruses were involved remained uncertain. As detailed in Section 2, BLAST (NCBI) and MEGA 6 were applied to align (Muscle algorithm) and compare the nucleotide sequence of T75 (GenBank: KT006374) to sequences from representatives of the B4, B5, and A9 coxsackieviruses, and echoviruses 9, 11, 19, 20, 26, and 27, as well as other SVDVs. Only viruses with a complete genome or with the maximum percentage of similarity in the regions encoding the compared proteins were selected (Table 1). The protein coding regions were determined based on the sequence of the CVB4 J.V.B., Benschoten strain (GenBank: X05690). The nucleotide sequence similarities were then calculated for each region in MEGA 6.

Notably, based on experimental data, the VP1/2A junction region was precisely determined by [45]. VP1 turned out to be nine nucleotides (three aa) shorter than previously predicted. Thus, the VP1/2A junction is at 3290/3291 n. with a SLVTT motif at the C-terminal end of VP1 in T75. Although computational analyses are useful in understanding genetic relationships, limitations like sampling bias and alignment artifacts can occur and these results should be further analyzed using wet-lab techniques.

In the BLAST analysis (NCBI), the maximum nucleotide homology was determined for sequences in the regions of precursors (P1, P2, P3) and for mature T75 proteins (Table 1). Viruses that circulated before 1975 were preferentially chosen for this analysis, but some sequences from the 1990s were also included. Many of the complete sequences for CVA9 were excluded since these viruses were isolated in the current century. Additionally, some sequences with high homology were excluded due to incomplete coverage.

CVB4 had the highest sequence similarity with the T75 virus in the 5′-UTR and in the region coding the P1 capsid protein precursor (85–94% and 85–90%, respectively). For the P2 precursor, the highest percentage of similarity (82%) was found with the early echovirus E1 from Egypt (strain Farouk, 1951, GenBank: AF029859) and type 11 echoviruses, which circulated in 1982–1989 in Russia and Finland. The T75 P3 precursor shows maximum homology (88%) with the early Italy/1/1966 SVDV strain and an 87% similarity with SVDVs isolated on the Eurasian continent from 1970 to 1976. It also has a high level of homology to echoviruses 9 and 11 (86%) which circulated in the USA and Europe from 1953 to 1989 [15]. It is of note that although percentage of sequence identity is a useful approximation of relation, it alone does not confirm evolutionary origin.

**Figure 3:**
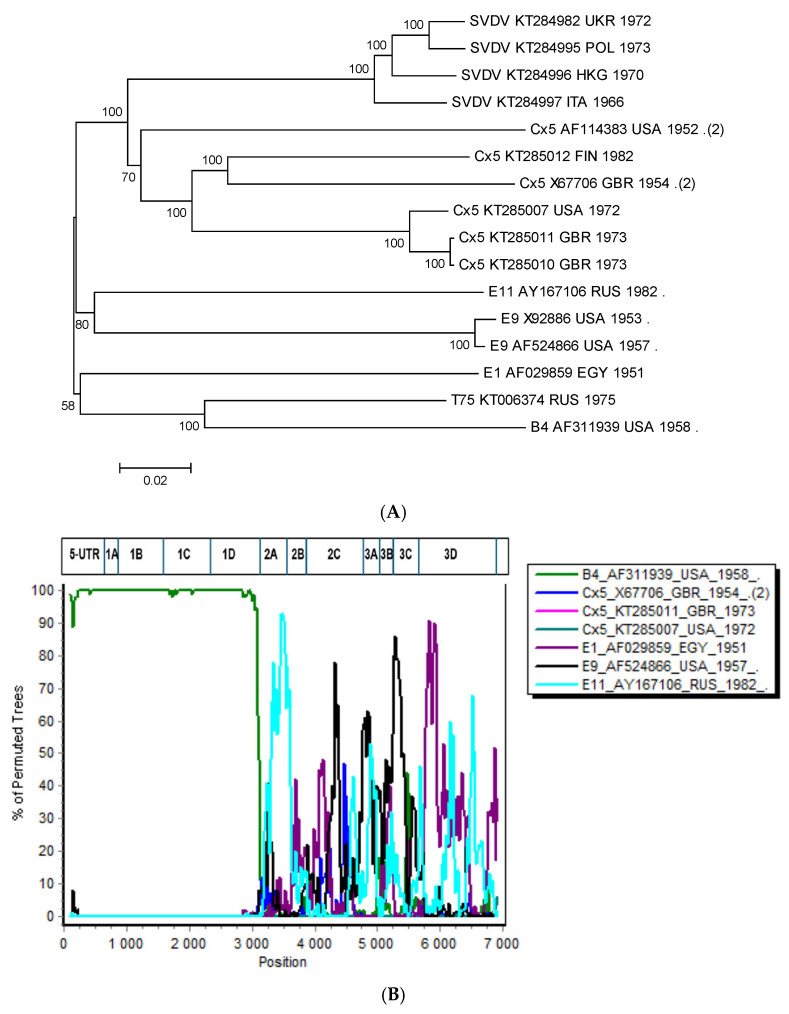
Genetic recombination analysis of the T75 strain (GenBank: KT006374). (**A**) Phylogenetic tree reconstruction by neighbor-joining statistical method with 1000 bootstrap replications in MEGA 6. (**B**) BootScan analysis with a window size of 200 and a step size of 20 was carried out in SimPlot (Version 3.5.1) for representatives of *Enterovirus betacoxsackie* species relative to T75 strain. Genome structure is detailed above the graph. Genomic nucleotide positions are shown on the *X*-axis, while the *Y*-axis shows the probability of cluster existence on the phylogenetic tree in % (see Figure 3A). The confidence level was 60% based on analysis of 100 replicates using the Kimura 2-parameter (K2P) model and neighbor-joining method. The legend shows strain nomenclature with accession number (in GenBank), ISO country code, and isolation year. Designations are SVDV: swine vesicular disease virus; B4: Coxsackievirus B4; Cx5: coxsackievirus B5; echoviruses: E1, E9, E11.

Analysis using SimPlot software (Version 3.5.1., Johns Hopkins University (Baltimore, MD, USA) was carried out to determine possible recombination events between the T75 virus and representatives of different groups (Table 1). Recombination analysis (Figure 3) revealed that the genomic 5′ region encoding capsid proteins was inherited by T75 from CVB4. The 3′ region encoding non-structural proteins was formed as a result of multiple recombination events between human enteroviruses. The main recombination breakpoint was in the 2A protein coding region [15]. In contrast, a breakpoint in the 2B protein coding region arose during recombination between two SVDV sub-lineages which circulated simultaneously in Italy in the first decade of the 2000s [19].

## 8. The Origin of SVDV from Recombination Events

Enteroviruses exist as a global gene pool, constantly evolving due to the exchange of genome fragments and mutational processes [6]. Recombination events have been shown between CVB4, CVB5, CVA9, and 17 serotypes of echoviruses [5,13], as well as between SVDVs themselves [13,19].

The CVB5 and SVDV-1 viruses have similarities in the capsid protein region, despite their different natural hosts [10,17,25,26]. Attempts to uncover the origin of the SVDV non-structural regions [10,11,12,13] were limited by the availability of only short genomic fragments (typically 400–800 n.), mostly in the 3BC and 3D regions. Additionally, for comparison only the VP1 SVDV-1 region was used. The data which was obtained supported the monophyletic recombinant origin of SVDV-1 from CVB5 in the capsid region, but it also speculated about an origin of the non-structural region from recombination of short regions from echovirus 9 (strain Barty) [10,11], or CVA9 [12] and other human enteroviruses, without strong evidence for the complete non-coding region. Although, it is impossible to reliably determine where and when the last common ancestor of CVB5 and SVDV-1 appeared, some scientists consider that the major center of CVB5 transmission was in China whereas the major center of SVDV-1 transmissions were in Italy [13].

To study the origin and evolution of enteroviruses, genomic regions without recombination breakpoints are usually used. These are regions of structural proteins (VP1) and conservative non-structural proteins (3C, 3D) [11,12,13,14].

Various studies have determined that the estimated time to most the recent common ancestor (TMRCA) for SVDV-1, based on VP1, was at approximately the same time (1948–1964) as the proposed CVB5 adaptation to pigs [11,13,14].

**Figure 4:**
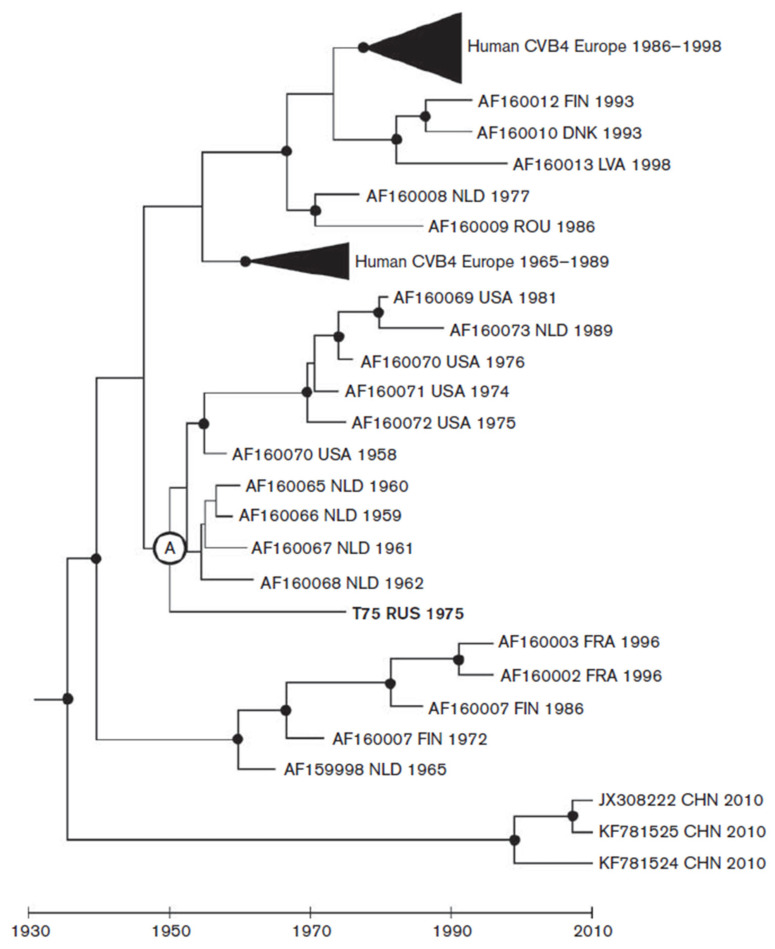
Phylogenetic tree of CVB4, including strain T75 (bold). The dataset contained 65 full-length VP1 sequences (924 n.). Phylogenetic analysis was performed using a Bayesian likelihood-based algorithm implemented in BEAST 1.7.5 [46]. The SRD06 substitution model was used with a relaxed log-normal clock. Analysis was run over 50 million generations and trees were sampled every 10,000 generations, resulting in 5000 trees. Trees were annotated with Tree-Annotator 1.4.8 using a burn-in of 1000 trees and visualized with FigTree 1.3.1. Bar: Time in years. Ages of nodes are indicated with circles. Node A had posterior probability values of 0.95. Reproduced from [14]. (Permission to reproduce the figure was obtained from Joe Kelly, Publishing Operations Lead, Microbiology Society, www.microbiologysociety.org).

In our previous work, analysis of 65 complete VP1 CVB4 sequences determined that date of the TMRCA of strain T75, and of other CBV4 strains, was in 1950 (Figure 4, node A), with a 95% probability density interval that the date was between 1945 and 1954. Therefore, the swine T75 virus most likely diverged from human CBV4 after 1945, but before its isolation in 1975 [14]. Thus, the ancestors for swine T75 and SVDV-1 emerged from human CVB4 and CVB5 viruses, respectively, in a close time interval (not earlier than 1945–1954 or 1948–1964, respectively).

Between 1975 and 1977 in the Tambov and neighboring regions in Russia, the T75-lineage virus affected more than 24,500 pigs. The presence of this virus was confirmed by partial VP1 sequencing of the one of the isolates, T77, which was sampled at the end of the epizootic in 1977. During the two years of circulation in the new pig host, four nucleotide mutations occurred in the short VP1 region (331 n.), resulting in two amino acid substitutions (C56Y, K98R; VP1 numbering according to T75) [15]. The total nucleotide substitution rate was found to be 0.0064 substitutions/site/year (s/s/y) for this region. This was slightly higher than the global CVB4 VP1 (0.00482–0.00520 s/s/y) [47] and for SVDV VP1 (0.00334 s/s/y [11] and 0.0039–0.0051 s/s/y [13]).

It is hypothesized that the swine T75 virus arose due to a rare, single event. The outbreak appeared suddenly in February 1975 in one pig farm, from where it spread to neighboring farms and regions in a short time. It was determined later that the spread occurred when piglets, which probably had sub-clinical infection, were transferred for fattening (pp. 79–90) [34]. A thorough investigation by veterinary services to find out the causes of the epizootic did not reveal the source of infection [34]. All food and ingredients received in the period preceding the epizootic, from the affected region and from abroad, were tested for the presence of the SVDV. Among them was maize imported from Romania, where CVA9 was present [6] (see Figure 2). Human enteroviruses were also widespread throughout Russia during that time. Unfortunately, there is no further information on this topic in open sources. From current knowledge, it can be hypothesized that the likely source of infection was non-disinfected human food waste from canteens that was then fed to pigs [34]. During the epizootic period, attempts were made to identify the pathogen among animal viruses, but human viruses were not evaluated.

Thanks to the precise actions of the veterinary services, the epizootic was stopped and the pathogen was eradicated. Nowadays, modern methods of molecular research allow reconstruction of unique events leading to the appearance and disappearance of recombinant enterovirus T75, which likely arose as a result of the cross-species transfer of the CVB4 recombinant common ancestor from humans to pigs with following adaptation.

## 9. Conclusions

Studies of the emergence and evolution of SVDVs hypothesize at least two independent events of cross-species transmission of human enteroviruses to pigs, which occurred due to multiple recombination events between human enteroviruses of different serotypes. Finally, two variants of SVDV (SVDV-1 and SVDV-2) emerged. The capsid protein region of SVDV-1 arose from CVB5, and SVDV-2 from CVB4. According to data from the literature and our own research, several human representatives of *Enterovirus betacoxsackie* species participated in the formation of the 3′ non-structural regions.

Transmission to a new host was accompanied by increased virulence of the recombinants, which caused numerous epizootics among pigs, worldwide. It took half a century to eradicate SVDV-1 in Italy and European countries, while SVDV-2 was suppressed in Russia within two years.

In this work, we have reviewed previous research and included previously unpublished data to together formulate a hypothesis of the origins of SVDV-2. As much of the previous research from the USSR is limited to internal theses and reports, there is still much to understand about the origins of this virus. It would be beneficial if future work sought to fully understand the recombination events which led to the virus responsible for the 1975 outbreak. Now, up-to-date techniques, like next-generation complete genome sequencing, phylogenetic analysis and access to various bioinformatic databases, allow identification of new emerging microbiological agents in a short period of time. However, it has been more than half a century since SVDV-2 first appeared in Russia (1975), and more is still being uncovered about the nature of this virus.

Currently, the issues relating to SVD have been resolved, as the virus is considered to be eradicated. However, the emergence of at least two virulent porcine variants from human enteroviruses, driven by recombination, adaptation and host transmission, remains an important lesson for researchers, particularly those in early-career stages, as novel viruses continue to arise.

## Figures and Tables

**Table 1:** Genomic similarity between the T75 strain [T75_KT006374_RUS_1975] and other enteroviruses (%).

Virus ^A^	Strain	Genomic Region and Its Location Relative to KT006374 (T75)														
		5′UTR	P1	P2	P3	VP4	VP2	VP3	VP1	2A	2B	2C	3A	3B	3C	3D
**Coxsackievirus B4**		1–743	744–3291	3292–5024	5025–7292	744–950	951–1733	1734–2447	2448–3291	3292–3740	3741–4037	4038–5024	5025–5291	5292–5357	5358–5906	5907–7292
AF311939_USA_1958	E2 [Edwards]	**94**	**90**	79	78	**92**	**90**	**91**	**90**	**80**	77	79	75	78	80	78
X05690_USA_1951	Benschoten	85	81	79	78	84	83	81	80	77	79	80	74	83	79	78
DQ480420_ITA_1999	Tuscany	85	81	79	78	85	83	81	80	77	79	80	75	83	79	78
KC558567_DNK_?	Cph9	-	85	80	79	85	85	85	84	**80**	78	80	78	74	79	79
KC558563_DNK_?	Cph5	-	85	80	79	84	85	85	85	79	79	81	78	77	79	79
**Coxsackievirus A9**																
DQ388926_ROU_1973	113/73/2	-	-	-	**^B^90**fr	-	-	-	-	-	-	-	-	-	**^C^**fr	fr
**Echoviruses**																
E1_AF029859_EGY_1951	Farouk	83.6/82	69	**82**	**86**	75	73	70	64	77	83	84	78	86	85	**89**
E9_X92886_USA_1953	Barty [Hill?]	83	64	81	**86**	76	68	63	59	76	76	**85**	**89**	86	**89**	84
E9_AF524866_USA_1957	Barty	84	64	81	**86**	75	69	63	59	76	77	**85**	**89**	86	**89**	84
E11_AY167106_RUS_1982	Mor/M/82	80	66	**82**	**86**	74	69	67	59	76	**86**	82	85	85	85	87
E11_AY167104_RUS_1987	Kar/87	83	67	**82**	**86**	75	70	65	59	77	82	**85**	83	84	87	86
E11_AJ577590_FIN_1989	FIN-0666	83	66	**82**	**86**	76	69	65	63	77	83	83	83	82	85	87
E11_AJ577594_ROU_1991	ROU-9191	83	67	81	85	76	70	66	63	76	81	83	79	86	86	87
E11_EF634316_SVK_?	D207	82	67	81	**86**	75	70	65	63	77	82	83	80	84	85	87
E19_AY302544_USA_1955	Burke	82	65	78	79	75	69	64	60	76	80	79	76	84	77	80
E19_AY167107_RUS_1981	K/542/81	81	65	79	84	77	71	65	58	78	75	80	75	82	83	87
E20_AY302546_USA_1956	JV-1	84	66	80	80	81	66	69	58	79	80	81	77	**89**	79	80
E26_AY302550_PHL_1953	Coronel	81	63	79	80	75	68	63	54	76	80	80	79	85	80	80
E27_AY302551_PHL_1953	Bacon	**89**	63	81	79	74	67	63	57	78	81	82	77	82	79	79
**SVDV**																
AY429470_HKG_1970	HK′70	**^D^**82/80	69	79	87	76	72	69	65	76	76	81	85	84	85	88
X54521_GBR_1972	UKG/27/72	81/81	70	79	87	77	72	70	66	75	76	82	85	83	86	88
D16364_JPN_1973	J1′73	81/79	70	79	87	77	72	71	65	75	76	81	85	84	85	88
KT284996_ HKG _1970	HKN/19/70	-/83.3	69	80	87	76	70	70	64	75	75	83	85	81	86	88
KF963276_HKG_1973	HKN-8-73	-/80	70	80	87	77	73	70	65	76	77	82	**86**	83	86	87
KF963277_HKG_1974	HKN-18-74	-/81	70	80	87	77	72	70	65	75	77	82	84	83	85	88
KF963278_HKG_1975	HKN-25-75	-/80	70	79	87	79	72	70	66	75	76	82	85	81	86	87
KF963274_HKG_1976	HKN-1-77	-/81	70	79	86	77	73	71	65	74	77	82	84	81	85	88
D00435_JPN_1976	H/3’76	82/80	70	79	87	78	72	70	65	75	77	81	85	84	85	88
KT284997_ITA_1966	ITL/1/66	-/84.6	69	79	**88**	77	71	69	64	75	76	82	**86**	80	88	**89**
KT284995_POL_1973	POL/1/73	-/80.4	68	79	87	77	71	68	64	75	75	82	85	83	86	88
KT284982_UKR_1972	USS/6/72,O72	-/82.1	68	79	87	77	70	69	64	75	75	81	86	83	86	88
EU151454_ITA_1992	Itl. 2-92	81/86	69	78	85	78	71	70	64	75	76	81	81	81	84	86
**Coxsackievirus B5**																
AF114383_USA_1952	Faulkner	83	70	80	79	75	72	70	65	**80**	78	81	74	81	79	80
Cx5_KT285007_USA_1972	4469/USA/72	-/85.6	69	82	**87**	77	72	70	62	74	80	**86**	**87**	83	85	**88**
X67706_GBR_1954	1954/85/UK	83	69	80	79	76	73	71	63	76	78	82	78	80	78	79
Cx5_KT285011_GBR_1973	8068/UK/73	-/84.6	69	82	86	77	72	70	62	74	80	**86**	85	81	83	87

Notes: -, no data; ?, unknown. ^A^ GenBank Accession number, ISO country codes, isolation year. ^B^ Fragment of 3CD region (5844-6459). ^C^ Partial fragment. ^D^ Percent similarity in the complete/partial 5′-UTR corresponding to fragments (1–739)/(439–739). Numbering relative to KT006374. The highest percentage of homology in each gene is designated in bold font and highlighted.

## Data Availability

No new data were created or analyzed in this study except for those that were not previously published for the reasons mentioned in Section 9.
